# The Clinical Overlap between the Corticobasal Degeneration Syndrome and Other Diseases of the Frontotemporal Spectrum: Three Case Reports

**DOI:** 10.1155/2007/218189

**Published:** 2007-08-22

**Authors:** Alberto Raggi, Alessandra Marcone, Sandro Iannaccone, Valeria Ginex, Daniela Perani, Stefano F. Cappa

**Affiliations:** ^1^Department of Neurology and NeurorehabilitationSan Raffaele Turro HospitalMilanItaly; ^2^Department of Psychology and NeuroscienceVita-Salute San Raffaele UniversityMilanItaly; ^3^Department of Nuclear MedicineSan Raffaele Scientific Institute and Milano Bicocca UniversityMilanItaly

**Keywords:** Corticobasal degeneration, frontotemporal dementia, nonfluent progressive aphasia, progressive supranuclear palsy, semantic dementia

## Abstract

The corticobasal degeneration syndrome has been suggested to be part of a complex of conditions (including the different subtypes of frontotemporal dementia and progressive supranuclear palsy), which reflect a spectrum of pathological substrates. This concept is supported by the frequent clinical overlap that can be observed among patients diagnosed with these conditions. We report three clinical cases, characterized by the overlap of the clinical features of corticobasal degeneration syndrome with, respectively, nonfluent progressive aphasia, progressive supranuclear palsy and semantic dementia. Current diagnostic criteria emphasize differences in clinical presentation, which probably reflect the preferential location of pathology in the early stages of disease. However, with disease progression, a considerable clinical overlap can be expected among the different syndromes. This concept should be extended not only to the cognitive and behavioural features of the frontotemporal dementia subtypes, but also to the movement disorders of corticobasal degeneration and supranuclear palsy.

## 1. Introduction

Corticobasal degeneration (CBD), together with progressive supranuclear palsy (PSP), frontotemporal dementia (FTD), parkinsonism linked to chromosome 17, postencephalitic parkinsonism, post-traumatic parkinsonism, parkinsonism-dementia complex of Guam, Alzheimer's disease, Niemann-Pick type C disease, and subacute sclerosis panencephalitis, is considered as a "tau pathology" [1,16,19]. The clinical diagnosis of CBD can be difficult, because of the clinical overlap with other neurodegenerative disorders, in particular PSP, when patients present with atypical manifestations, such as asymmetric onset, mild oculomotor impairment, involuntary limb levitation resembling the alien-limb phenomenon, and focal dystonia. When CBD manifests itself predominantly with language symptoms, there may be diagnostic confusion with non fluent progressive aphasia. Furthermore recent clinical and pathological studies have suggested that the behavioural variety of FTD and CBD may show clinical and pathological overlap [5,11,12,18]. In contrast, the overlap between semantic dementia (SD) and CBD has not been reported. This may be due to the fact that the former is often associated to an ubiquitine

ISSN 0953-4180/07/$17.00 © 2007 – IOS Press and the authors. All rights reserved positive tau negative pathology [13,23], while the latter is usually correlated to a tau positive pathology [19].

We report three cases in which the clinical diagnosis of corticobasal degeneration syndrome (CBDS) was problematic, because of the presence of clinical features resulting in an overlap with other conditions within the FTD-PSP-CBD spectrum. The first was characterized by the overlap of signs and symptoms of FTD and CBD and by a relatively fast progression, the second could be characterized as clinically probable PSP without clear exclusion criteria for CBD; in the third patient the typical features of SD were present together with severe apraxia, which is typical of CBD.

## 2. Case reports

### *2.1. Case report 1*

The patient was a right-handed man, with eight years of education. He came to our observation at the age of 80, three years after the onset of balance and gait disorders and speech abnormalities.

There was no family history of neurologic and psychiatric diseases and no history of alcohol abuse. His medical history was positive for mild hypertension and a very recent diagnosis of prostate carcinoma and mild bilateral deafness. He had had a right carotid endarterectomy five years before and a bilateral hip arthroplasty twelve years before. The initial complaint was a progressive disorder of speech articulation. When observed for the first time, he already had a severe dysarthria. The speech disorder was followed after six months by a progressive impairment of language production, characterised by word finding difficulties, simplification of sentence structure and morphological errors. The motor features at presentation were left upper limb clumsiness, associated to balance and gait disorders. At the beginning he could walk with help. At the time of examination there was a difficulty in initiating movements, in rising, standing, and walking, and the automatic sequence of complex movements was lost. The gait was almost impossible, shuffling and slow, with very short steps (never more than three or four with a caregiver's help). He showed a discrepancy between the severe disability when attempting to walk and the preservation of the leg movements when lying and sitting. He started to show a motor neglect of the left limbs, which was more severe for the arm. Moreover, he became irritable and was not able to control his emotions. There was a fast progression of motor (limb clumsiness, speech abnormalities, gait disorder), neuropsychiatric (irritability) and cognitive (apraxia, alien-limb phenomena, frontal-lobe-release signs) symptoms during the three-year period.

At the time of the first evaluation the neurological examination revealed: dyspraxia of speech (severe dysarthria and dysprosody) with non-fluent aphasia; ideomotor apraxia of the left limb with partial alien-limb phenomena; left-side bradykinesia, diminished left hand finger movements, asymmetric (left $>$ right) mild rigidity, moderate left upper limb dystonia, mild action and postural tremor of the left hand; Myerson's sign, hooking response (forced grasping), bilateral palmomental reflex of Marinesco-Radovici; the sensory examination was entirely normal; he had lost the ability to use the lower limbs (left $>$ right) for walking, although there was no demonstrable sensory impairment or motor weakness (gait apraxia).

A language assessment performed when the patient came for the first time to our observation revealed a dysarthria associated to dysprosodia-dysphonia. Language production was very limited, with a few verbal-semantic and phonemic paraphasias and severely agrammatic/paragrammatic production. Repetition and reading of words and non-words was impaired on a specialized examination (B.A.D.A.) [17]. Words repetition: 24/45 (cut-off: 45). Non-words repetition: 9/36 (cut-off: 36). Words reading: 71/92 (cut-off: 90). Non-words reading: 27/45 (cut-off: 43). The patient showed also an impairment in word writing under dictation, with a total score of 38/46 (cut-off: 44). Oral and written naming for both objects and actions was relatively preserved with the following scores: oral object naming = 28/30 (cut-off: 28), oral action naming = 26/28 (cut-off: 26); written object naming = 21/22 (cut-off: 20), written action naming = 20/22 (cut-off: 20). Visual comprehension was conserved: visual word picture matching task for objects = 40/40 (cut-off: 38), visual word picture matching task for actions = 18/20 (cut-off: 18). In the auditory comprehension tasks, the patient had mildly impaired scores: auditory word picture matching task for objects = 36/40 (cut-off: 38), auditory word picture matching task for actions = 17/20 (cut-off: 18). Visual and auditory sentence comprehension subtests scores were normal: auditory sentence picture matching task = 60/60, written sentence picture matching task = 43/45. On the Pyramids and Palm Trees Test (PPTT) [7], assessing semantic memory, he had a score of 30/30.

The performance in memory, executive functions and visuo-spatial abilities tests was within normal limits.

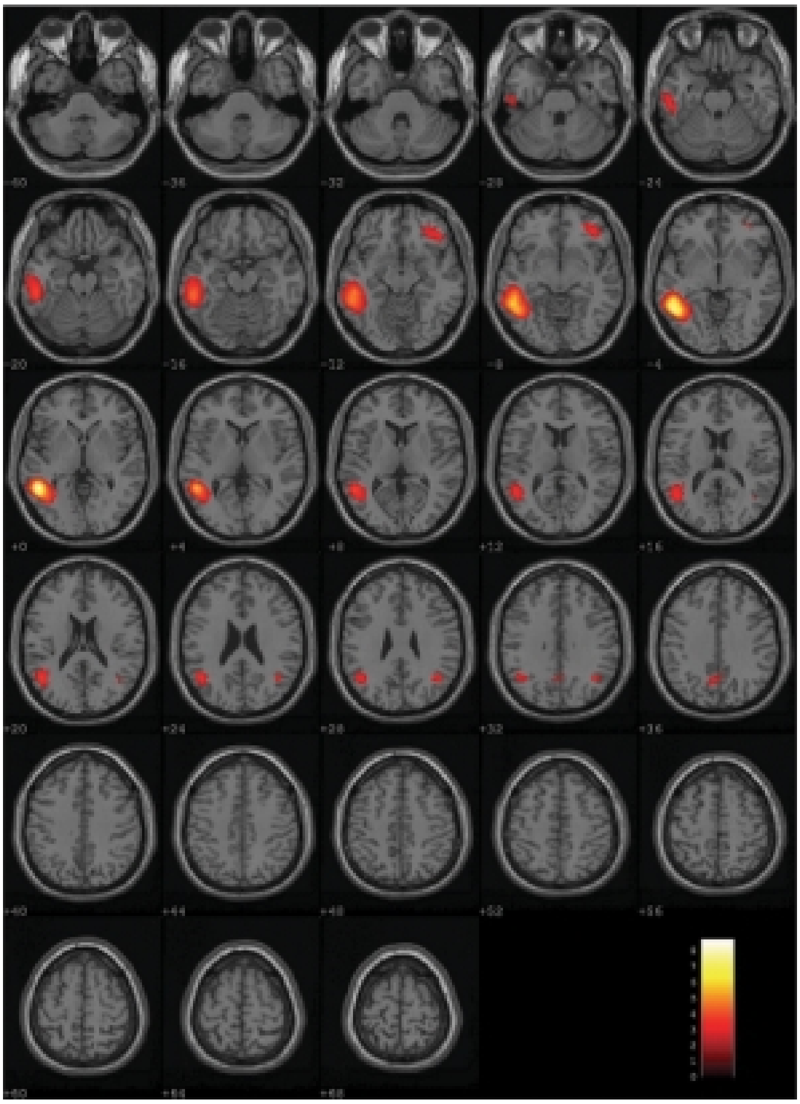

Fig. 1. Case 3 Statistical parametric maps (SPM99) showing: (A) hypoperfusion involving the left temporal lobe.

After a 6-month period, he could not be given the full test battery because he was completely anarthric, and comprehension had deteriorated. Praxis functions assessed at the time of the second examination with the De Renzi, Motti & Nichelli Test [2] resulted in a total score of 57/144 (Right side: 41/72. Left side: 16/72).

The electroencephalogram (EEG) and electromiography (EMG) were normal. Brain Magnetic Resonance (MR) demonstrated mild vascular changes in the pontine region, in the subcortical white matter and at the capsulo-lenticular level bilaterally. Brain Single Photon Emission Computed Tomography (SPECT) showed reduced mesial frontal perfusion bilaterally.

### *2.2. Case report 2*

The patient was a 68 years old right-handed woman with eight years of education. The family history was negative for neurologic and psychiatric diseases. In the medical history there was mild hypertension. She had been submitted to colecistectomy for choledocholithiasis some years before. At the beginning of the sixth decade of life she started to complain about "a constant aching of her arms". At the age of sixty-four, she began to show a progressive dysarthria with nasal voice, which became very severe in the course of the last year. After one year she developed an unsteady gait, with frequent, sudden falls, particularly backwards. Her sons described an almost constant akathisia and a tendency to stand up impulsively, without concern for the context and the risk of her act. Cognitive and psychiatric deficits date from the age of sixty-six. The caregivers reported that she became apathetic, neglecting self care, and was disorientated in time, and sleepless. She attempted suicide by ingestion of zolpidem tablets. At admission, she had severe dysarthria, a balance and gait disorder, cognitive impairment, and loss of voluntary eyes movements.

The neurological examination revealed: severe dysarthria, loss of voluntary vertical eye movements (early stage of vertical supranuclear gaze palsy), reduction in facial expression, bradykinesia, left-side bradykinesia, asymmetric (left $>$ right) mild rigidity, brisk tendon reflexes without Babinski signs; Myerson's sign, bilateral palmomental reflex of Marinesco-Radovici. The sensory examination was entirely normal. Moreover, there was a left limbs clumsiness and involuntary levitation, propensity to fall backward, and gait apraxia.

The language evaluation showed normal performances except for speech articulation. Language production was tested with Aachen Aphasia Test (AAT) [8]: word and sentence repetition = 149/150 (normal), reading and writing on dictation = 88/90 (normal), object and action naming = 111/120 (normal); comprehension was tested with B.A.D.A. [17] with the following scores: visual word picture matching task for objects = 40/40 (cut-off: 38), visual word picture matching task for actions = 20/20 (cut-off: 18), auditory word picture matching task for objects = 39/40 (cut-off: 38), auditory word picture matching task for actions = 19/20 (cut-off: 18), auditory sentence picture matching task = 58/60, visual sentence picture matching task = 43/45.

A significant deficit of praxis was observed, with a total score on the De Renzi, Motti & Nichelli Test [2] of 91/144 (Right side: 47/72. Left side: 44/72).

EEG and EMG were normal. The brain MRI demonstrated mild ischemic changes in the subcortical white matter. Brain SPECT showed bilateral parietal hypoperfusion.

### 2.3. *Case report 3*

The patient was a 56 years old right-handed woman with no family history of neurological and psychiatric diseases. Her medical history was negative. At the age of fifty she started to show mood and behavioural disorders: melancholy, inclination to repeat manners and assertions, frequent oversights. The disturbance was progressive. After three years she had lost self-care concerning hygiene, cosmesis and clothing. She became apathetic and depressed. The social withdrawal grew worse. At the age of fifty-six, the patient was no more able to take care of herself, the family and the house. She became dependent in many activities of daily life. For the first time, her relatives noted she had a language disturbance.

The neurological examination revealed: "closing in" sign; deficit of language and praxis functions (described below); normal cranial nerves functions; normal motor tone and strength, brisk tendon reflexes without Babinski signs; normal coordination; absence of abnormal movements; normal sensory examination; normal station and gait. She appeared elated and fatuous, and was anosognosic.

The evaluation of spontaneous language showed fluent speech without abnormalities of articulation, frequent anomias, semantic paraphasias and stereotypies. The main feature was a severe disorder of word and sentence comprehension. Language was formally assessed with the B.A.D.A. [17]: she was impaired in oral object naming = 15/30 (cut-off: 28), oral action naming = 17/28 (cut-off: 26). Naming was also assessed by the Boston Naming Test [29] in which she could give 6 correct answers out of 30 items. Also comprehension was pathological: auditory word picture matching task for objects = 30/40 (cut-off: 38), auditory word picture matching task for actions = 15/20 (cut-off: 18), visual word picture matching task for objects = 27/40 (cut-off: 38), visual word picture matching task for actions = 12/20 (cut-off: 18). On the Pyramids and Palm Trees Test (PPTT) [7], assessing semantic memory, she had a severely impaired score (10/30).

She also has a severe melokinetic and ideomotor apraxia on the De Renzi, Motti & Nichelli Test [2] with a total score of 69/144. Right side: 41/72 (27/36 of symbolic gestures and 14/36 of non symbolic gestures). Left side: 28/72 (19/36 of symbolic gestures and 9/36 of non symbolic gestures).

The memory evaluation shows a very mild impairment that could be attributed to defective understanding of instructions.

The EEG, EMG, motor and somatosensory evoked potentials were normal. The brain MRI demonstrated diffuse cerebral atrophy. Brain SPECT showed a focal reduction of perfusion in the left temporal lobe (Fig. 1).

## 3. Discussion

Recent studies have indicated that FTD and CBD show clinical and pathological overlap [5,11,12,18].

The three cases described here represent examples of the diagnostic uncertainties at the clinical level, which may be related to this pathological overlap. In Case 1 we find symptoms and signs compatible with a diagnosis of CBD [15], but also of nonfluent progressive aphasia (PNFA). There are now several reports of CBD pathology in patients diagnosed as PNFA [10]. This case demonstrates that PNFA and CBD can appear in the same patient at different stages of the disease, probably in relation to the progression of anatomical damage. The relationship of the different clinical presentations has been strengthened by the discovery of chromosome 17 linkage in families manifesting phenotypic variations [4]. The gene for tau protein associated to neurofibrillary tangles is located in this chromosome region. In some instances of FTDP-17, affected members in a family have shown the typical CBD phenothype [3,9,21,26], demonstrating a direct causal association between the two conditions. Another peculiarity of Case 1 is the association between the SPECT findings, showing reduced mesial frontal perfusion bilaterally, and gait apraxia, which can be defined as a loss of ability to properly use the lower limbs in the act of walking that cannot be accounted for by demonstrable sensory impairment or motor weakness. The combination of bradykinesia and ataxia in frontal lobe disease, like in our case, can be explained by interruption of the connections between motor, premotor, and supplementary motor cortex and other subcortical motor areas, such as the cerebellum and basal ganglia [20].

The diagnostic criteria for PSP seem to be respected [14] in Case 2. However, in the same case we found clinical manifestations that are typical of CBD, such as left limbs apraxia. As for the neuroimaging finding (SPECT), parietal hypoperfusion is more common in CBD than in PSP. A clinical overlap with supranuclear gaze palsy has already been described in one case that was later neuropathologically confirmed as CBD [25]. Pathological overlap has also been reported [24]. Common to both tauopathies is that isoforms of four-repeat tau due to splicing of exon 10 define the tau filamentous aggregates [28]. This is in contrast to other tau disorders, such as Pick's disease, which are characterized by three-repeat tau aggregates [22]. Additional evidence for a link between PSP and CBD is the finding that both disorders are homozygous for the H1 tau haplotype [6]. Furthermore, in some families with parkinsonism linked to defined mutations of the tau gene (FTDP-17), involved relatives have presented with the PSP phenotype [3,18]. Similarly, in familial multisystem tauopathy with presenile dementia, a heterozygous splice donor site mutation of tau gene has been identified, leading to a clinical phenothype and brain thau pathology reminiscent of CBD and PSP [27]. To summarise, although CBD and PSP can usually be separated both clinically and pathologically, the degree of clinicopathological and genetic overlap suggests that they represent different phenotypes of the same disorder, with differences possibly reflecting the genetic background. Therefore, it cannot be excluded that PSP and CBD are distinct nosological entities occurring in subjects with similar genetic predisposition [24].

The third case shows the least expected overlap. The severe, progressive disorder of naming and single word comprehension which is typical of SD was present, together with a severe, asymmetric apraxia, with melokinetic and ideomotor features, which is typical of CBD. There is accumulating evidence that SD is usually associated to ubiquitin positive tau negative pathology, while primary progressive aphasia and CBD/PSP syndrome are often found in patients with tau positive pathology [13]. Therefore a clinical overlap between SD and CBD is unexpected. We cannot exclude an atypical presentation of Alzheimer's Disease (AD). Given the preservation of memory and the severity of apraxia, only AD pathology in an atypical distribution could underlie the clinical picture observed in this subject. The patient however had none of the typical motor features of CBD. The SPECT pattern was asymmetric and involved the left temporal lobe. The answer to the diagnostic riddle exemplified by this case may come only from further follow-up.
